# Microbes Associated to Dyer’s woad (*Isatis tinctoria* L.): Pigment Extraction, Dyeing and Cultivation with Non-toxic Inputs. A Review

**DOI:** 10.1007/s00284-025-04515-4

**Published:** 2025-09-29

**Authors:** Pirjo Yli-Hemminki, Juha-Matti Pihlava, Johanna Leppälä, Marjo Keskitalo

**Affiliations:** 1 Natural Resources Institute Finland (Luke), Jokioinen, Finland; 2 Natural Resources Institute Finland (Luke), Rovaniemi, Finland

## Abstract

Dyer’s woad (*Isatis tinctoria* L.) is a biannual plant cultivated mainly for its leaves, which are source of precursors of natural blue pigment known as indigo. Pigment extraction and dyeing with indigo have traditionally been mediated by bacteria. Specifically, indigo-reducing bacteria convert the pigment to its soluble form, which then drifts to the water-immersed textile material in a vat dyeing process. Upscaling these microbial processes to an industrial scale, requires an understanding of how the appropriate bacterial community is applied and maintained in an anoxic, alkaline and hot vat system. Bacteria enter the system with leaf material and may originate from the soil. Therefore, bacterial communities, which have been extensively studied in Japanese indigo dyeing baths usually differ from those derived from European woad. Currently, characterised indigo-reducing bacterial isolates are available and recombinant microbes for indigo biosynthesis have been developed to replace synthetic and often toxic chemicals in the blue dye industry. Woad is defending its place in crop rotation, breaking monoculture as a functional allelopathic plant or as a nutrient scavenging catch crop, even in northern latitudes. High-yielding cultivars can be introduced into crop sequences, and indigo can be extracted on the farm to generate additional income for farmers’ cooperatives.

## Introduction

Today, natural plant dyes are used in the textile industry to improve the sustainability of products in environmentally conscious markets [1]. One of the most desired colours in textile dyeing is indigo blue. Traditional artisanal knowledge of plant indigo pigment extraction and vat dyeing with bacterial processes has driven research to improve the controllability of bacterially implemented dyeing techniques, especially in Japan, as reviewed by Aino et al. [2]. The consumption of renewable, natural dyes may increase, as their environmentally friendly production and application techniques are further developed to reach modern industrial scale. This review summarises original research and review articles on microbes and processes specifically associated with the plant Dyer’s woad (*Isatis tinctoria* L.), or woad for short. Previously, the methods used to reduce and dissolve indigo for dyeing have been revied by Blackburn et al. [3], and various recipes for woad fermentation vats have been experimentally tested by Hartl et al. [4]. Previous review articles about chemical and biological properties of indole glucosinolates [5–7], brassicas chemicals for pest management [8, 9], and the therapeutic potential of woad phytochemistry [10] have been incorporated to this review, insofar as woad was mentioned, especially to supplement the understanding of woad’s functionality in cultivation systems. The motivation for the review stems from the need of farmers and other practitioners to know what is required to run and scale up woad cultivation and dye production and how research could support low-input, low-technology and low-cost traditional applications. Biocolourants can add value to agricultural production. Production of natural alternatives for synthetic dyes by small and medium sized enterprises in rural areas could help to move towards a bio- and circular economy. Woad (*Isatis tinctoria* L.) is a biannual plant, which can be successfully cultivated in northern latitudes, while the other blue dye plants require warmer climates. Woad and other cruciferous plants are also known for their bioactive secondary metabolites, which can affect soil microorganisms by acting antagonistically on ammonium-oxidising bacteria and plant pest nematodes. As such, they can be used in crop rotations as functional plants, even if they are not intended for food or feed production. A better understanding of the microbial processes involved not only in indigo extraction and dyeing, but also in woad cultivation, could reduce the need for inputs and improve the profitability of farming. As a result, natural processes can replace toxic chemicals in farming and dyeing. These studies can also support the development of woad-derived dye production in northern latitudes. The aim of this review is to highlight microbially mediated processes associated to woad cultivation and dye processing. However, the search strategy for the review revealed that the bacteria associated with the extraction and reduction of plant indigo have recently been studied particularly in Japan, in the last decade, supporting the traditional uses of the dye as part of peoples’ cultural heritage. Emerging research focuses on the biotechnology of indigo pigment production and non-toxic reductants which are referred in separate paragraph. Publications for this review were searched in Web of Science and Google Scholar using the keywords: dyer’s woad, woad, Isatis tinctoria AND bacteria, microbes. More specific searches were made for: indigo-producing bacteria and for woad AND indigo reduction, nitrification inhibition, glucosinolates and arbuscular mycorrhizal fungi.

### Natural Plant Indigo

For thousands of years, dye plants have been collected from nature and later introduced into cultivation in Asia and Europe. Natural blue can be derived from the plants that produce indigo pigment precursors in their leaves. There are many indigo-bearing plants, such as the perennial Chinese rainbell *Strobilanthes cusia* (Acanthaceae), but the main sources are true indigo *Indigofera tinctoria* in the Fabaceae family, Japanese indigo *Persicaria tinctoria* (also known as *Polygonum tinctorium*) in the Polygonaceae family, and woad in the Brassicaceae family. Woad has been successfully cultivated in temperate and northern parts of Europe, including Finland. Woad is not an edible plant, and thus its cultivation should not compete with food production if it is grown in crop rotation and on marginal land.

### Biotechnological Alternatives in the Blue Dye Industry

The synthesis of the dye indigo from the by-products of the petroleum and coal tar industries, requires the usage of many environmentally hazardous chemicals [11]. At present, the production or reduction of indigo by biotechnological processes are well-established on experimental scale [12]. The production of indigo by bacterial strains with suitable mono- and dioxygenase enzymes, has been investigated as an alternative process, as the bacteria could use recycled raw materials in the production [13]. The bacteria are often aromatic hydrocarbon degraders, such as naphthalene- and toluene-degrading *Pseudomonas putida strains* [14]. Recombinant *Escherichia coli* carrying the genes for naphthalene degradation can produce indigo at high rates from media containing naphthalene, tryptophan and even glucose [13]. In this process, bacterial redox enzymes oxidise indoles to indoxyls, which then dimerise into indigo. As a step forward, the chemoenzymatic production of indican and its photolytic or enzymatic oxidation can eliminate the reduction step in yarn dyeing [15].

Dyeing fabrics with indigo requires its reduction to water-soluble leuco-form in a vat, and the industry uses large quantities of toxic hydrosulphides in this process. Sodium hydrosulphide causes various environmental problems due to its toxic reactions in waterways [3]. Also, it cannot be recycled from wastewater or reused in the reduction process [16]. Patra et al. [17] have developed a method that works at room temperature, using bacterial cell lysate of thermophilic bacteria from hot springs and sodium hydrosulphite for vat dye reduction. The main aim of recent research has been to develop methods for reducing indigo with, for example, glucose, electrochemical reaction or ferrous sulphate, which are more environmentally friendly [18]. Bacteria can initiate the reduction of indigo. The unreliability of spontaneously occurring bacteria in vat dyeing has led to the isolation and characterisation of indigo-reducing bacteria (Tables 1 and 2). Understanding the requirements of these bacteria may allow the development of their application on an industrial scale.

**Table 1:** Comparison of chemical and microbial reactions leading to indigo formation and reduction

Indigo formation		
Chemical reactions at standard treatment	Corresponding microbial reactions	References
Immerse woad leaves to hot water (60–80 °C) at low pH 3.5 to release isatan B molecules to solution. Cool the water quickly (25 °C) and adjust pH > 10 by calcium hydroxide. Indoxyls now separate from the glucose moieties. Aerate for dimerization of indoxyls to form indigo, which now precipitates. Sediment the indigo with citric acid	Early-stage aerobic bacteria consume oxygen from the stagnant warm water where leaf material is immersed. Isatans are enzymatically hydrolysed to glucose and indoxyl moieties: β-glucosidase enzymes in the *P. tinctorium* mesophyll cells chloroplasts produce indoxyl and glucose through hydrolysis reaction. *Paenibacillus* sp., *Bacillus megaterium* glycoside hydrolase can cleave indicant, *Alcaligenes faecalis* enhances indigo production by utilising sugar from indole as a sole C source. Indoxyls dimerize in oxic conditions to form indigo, which precipitates	[3, 18–22, ]

Indigo reduction in a vat		
Chemical reduction	Bacteria-induced reduction	References
Reduction potential of − 600 mV is required for indigo reduction. Added sodium hydrosulphide at 55 °C reduces indigo to its soluble leuco state. Other reductants are glucose, electrochemical reaction and Fe-sulphate Quinones interact with indigo particles and make them more easily reduced (1:400 at 65 °C, [18])	Bacteria exhaust oxygen and fermentation produces low reduction potential, − 600 mV needed for indigo reduction. The bacteria need simple C-sources, which are produced by fermenters. Bacteria (e.g. *Clostridium isatidis, Virgibacillus* spp., *V pantothenticus*) reduce indigo to its colourless soluble *leuco* form with the help of an intrinsic protein which interacts with indigo particles (smaller particles are better). Add Ca-hydroxide to pH > 10 to dissolve *leuco* indigo. Also, bacterial cell lysate with sodium hydrosulphite have been used as catalyst to reduce vat dyes to *leuco* form at room temperature	[3, 17, 18, 23]

**Table 2:** Bacteria that have been identified and/or characterised from woad vats and from Japanese indigo vats in dyeing processes

Microbes associated to woad vat	Characteristics	References
*Clostridium isatidis, C. perfringens, Bacillus* sp.	Gram-positive, endospore forming, anaerobe, moderately thermophile	[23–25]
*Geobacillus palidus, Ureibacillus thermosphaericus, Bacillus pallidus* enrich in couching process, while *Bacillus thermoamylovorans* enriches in the vat	Aerobe thermotolerant, thermophilic	[2, 3]
DGGE (> 97% similarity to reference): *Paenibacillus lactis, Sporosarcina koreensis, Bacillus licheniformis, B. thermoamylovorans*, and (< 97% similarity to reference) *B. thermolactis, B. pumilus, B. megaterium*. Pyrosequencing results: *Clostridium ultunense, Tissierella* spp., *Alcaligenes faecalis, Erysipelothrix* spp., *Enterococcus* spp., *Virgibacillus* spp., *V. panthothenicus*	Aerobe, anaerobe, alkaliphiles, halophiles, thermophiles, photosynthetic, lactic acid bacteria, some degrade indigo (*Bacillus pumilus, B. licheniformis*)	[20]

Microbes associated to Japanese indigo vat	Characteristics	References
Transition of bacterial communities in genus-level from *Halomonas*-dominance to *Amphibacillus, Clostridium* and *Oceanobacillus* in the early stage of the process, and to *Alkalibacterium psychrotolerans, A. iburience, A. indicireducens* in the late-stage vat aged for ten months	Facultatively anaerobes, alkaliphiles, thermophiles, lactic acid producing bacteria	[26]
*Bacillus alkaliphilus, B. fermenti, Alkalibacterium psychrotolerans, A. iburience, A. indireducens, Amphibacillus iburiensis, Oceanobacillus indireducens, Fermentibacillus polygoni, Polygonibacillus indicireducens, Paralkalibacillus indicireducens*	Gram-positive, alkaliphiles, facultative anaerobes, straight motile rods	[20, 27–29]
*Alkalibacterium, Pseudomonas*	Facultative alkaliphiles, thermophiles 50 °C, anaerobes in fermentation liquor aged 6 years	[30]
*Bacillus cohnii, B. rigiliprofundi, B. pseudofirmus, Polygonibacillus indicireducens, Anaerobacillus arseniciselenatis, Alkalibacterium iburiense, Amphibacillus indicireducens, A. xylanus, Oceanobacillus oncorhynchi*	Isolates recovered using wheat-bran and sukumo-based hydrolysate media, pH 10	[31]
*Alkalibacterium, Amphibacillus, Tissierellaceae, Proteinivoraceae, Anaerobacillus, Polygonibacillus*	Indigo-fermentation fluids aged 6, 9, 10, 11 and 14 months	[2]
*Alkalibacterium, Amphibacillus, Polygonibacillus, Bacillaceae, Anaerobranca, Polygonibacillus*	High pH, obligate anaerobes, aerotolerants	[32, 33]
*Firmicutes, Proteobacteria, Actinobacteria, Bacillaceae, Bacillus cohnii, B. taeanensis, Alkaliphilus oremlandii, Mogibacterium neglectum, Alkalihalobacillus alkalinitrilicus, Tissierellaceae, Polygonibacillus indicireducens, Erysipelotrichaceae*	Sukumo treated with *Indigofera tinctoria* leaf powder. Facultative anaerobes, oblicate anaerobes, aerotolerants	[34]

*DGGE* denaturing gradient gel electrophoresis

### An Overview of Plant Indigo Formation and Reduction

**Fig. 1:**
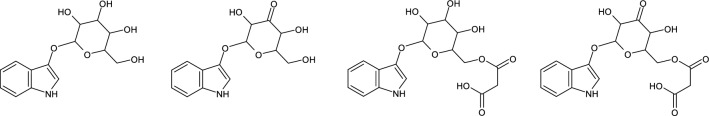
Chemical structures of the main precursors from left to right: indican (C_14_H_17_NO_6_), isatan B (C_14_H_15_NO_6_), isatan C (C_17_H_19_NO_9_), and isatan A (C_17_H_17_NO_9_)

Indigo does not occur in plant leaves as such, but as its precursor molecules, indoles, which form indigo in controlled steps. Indole is an intermediate common to the biosynthetic pathways of both indigo precursors and L-tryptophan, which in turn is a precursor of the plant hormone auxin (indole-3-acetic acid, IAA). The formation of indoxyl is therefore a secondary reaction in the plant [35]. It has been speculated that the many secondary indolic compounds in the plant are related to pest defence [36]. Indole-derived nitrogen-containing indoxyls with sugar moieties, i.e. indican and isatans A, B and C, are known precursors of indigo (Fig. 1). Isatan B is the major precursor that accumulates in the vacuoles of woad leaves [35, 37].

Woad´s indigo precursor amounts vary highly. The differences in plant´s phenotype and cultivation conditions can influence the production of these secondary metabolites [38, 39]. Freshly harvested young woad leaves contain higher concentrations of indican and isatans than older leaves (24% and 14% DW, respectively, [40]).

**Fig. 2:**
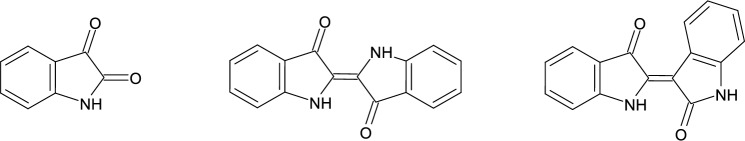
Chemical structures of isatin (C_8_H_5_NO_2_), indigotin (C_16_H_10_N_2_O_2_) and indirubin (C_16_H_10_N_2_O_2_)

Woad leaves cannot be dried as such for storage, as the precursors become inaccessible as they react within the plant tissues [37]. There are two methods for extracting indigo from the harvested plant biomass: (1) hot water extraction from fresh leaves and (2) the traditional slow processes that preserve the leaves first (Table 1) [11]. The traditional method is discussed later in Chapter 2. In the hot water extraction, the indigo precursors, isatans, are released from fresh, washed leaves into the hot water (60 °C), which melts the surface wax of woad leaves. The precursor molecules cannot be stored for long in this liquid as they are very sensitive to oxygen and light. As an attempt at storage, isatan B has been shown to be more stable at pH 3.5 [22]. In the next step, free indoxyl is cleaved from the isatans by hydrolysis of the sugar moieties at pH > 10 at room temperature. Oxidation of the liquid leads to dimerisation of two indoxyl molecules via acid *leuco*-indigo to indigotin, which then precipitates as the blue dye indigo (Fig. 2). Dried dye flakes can be stored indefinitely.

Newly formed woad indigo is susceptible to impurities and contaminants originating from the plant material itself and from the soil [41]. The purity of plant indigo is much lower, 20–60% for woad indigo compared to the synthetic dye (> 90%), due to the different efficiency of the extraction method (reviewed by [18]). Approximately 40% of the indoxyl moieties may be lost to impurities during the extraction. An undesirable side reaction in the process is the over-oxidation of indoxyl to isatin, which condenses with indoxyl to form the red pigment indirubin (Fig. 2).

**Fig. 3:**
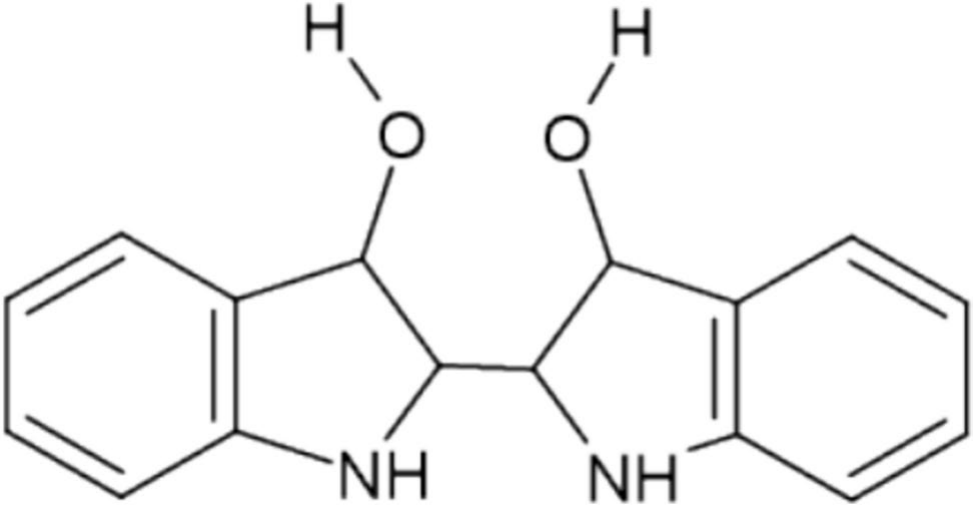
Chemical structure of leucoindigo

For textile dyeing, insoluble indigo must be reduced to its soluble *leuco* state by adding a reducing agent at pH 11 in warm water at 50–55 °C in a process called vat dyeing (Fig. 3). The reduction process changes the chromophore of the molecule to a pale yellow [3]. The high pH helps to keep the leuco indigo in solution and thus able it to enter to the fibre matrix immersed in water. Once the fabric is removed from the vat and exposed to air, the leuco indigo molecules are oxidised back to the original parent pigments, indigo, turning the fabric blue.

Indigo flakes are a storable and tradable form of the dye [4]. The conditions for the extraction of indigo precursors from leaves and their subsequent conversion to indigo have been tested, optimised and standardised also for farm-scale production [22]. Hot water extraction is simple and robust, but energy intensive procedure. Methods based on traditional processing of woad leaves, are similar to fermentation and their management requires craftsmanship.

## Origin and Role of Bacteria in Indigo Reactions

### Bacterial Reactions in Traditional Indigo Extraction and Dyeing

The traditional European woad leaf processing method shares some common features with the Japanese indigo processing, *sukumo*, such as ‘couching’, and they are sites for several microbially driven reactions [2]. Sukumo is a method of preserving Japanese indigo leaves by composting them for fermentation [33]. The methods are not directly interchangeable between Japanese indigo and woad due to differences in the leaf material of polygonum and cruciferous plants.

**Fig. 4:**
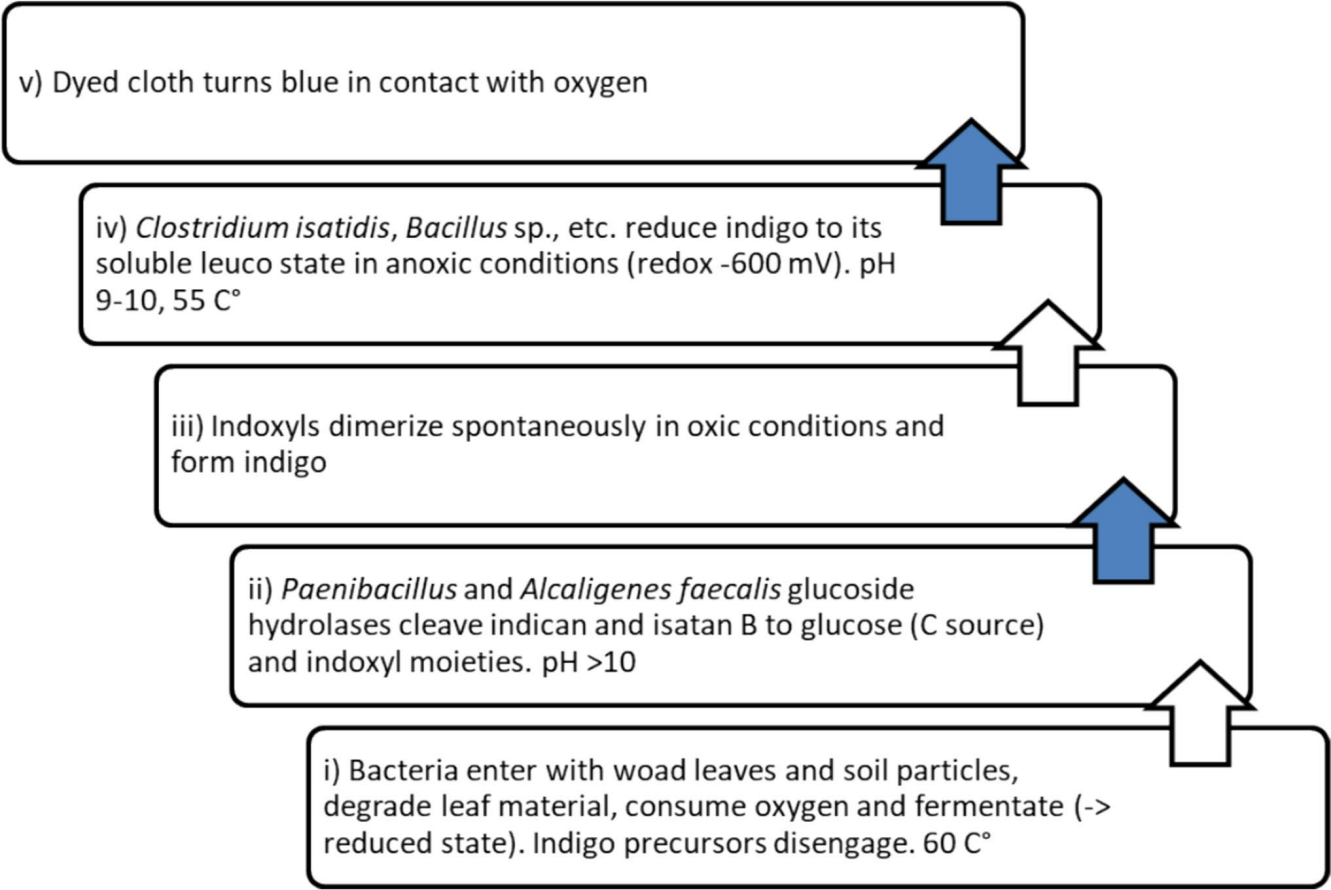
Schematic illustration of the bacterial processes involved in the formation (i, ii) and reduction (iv) of indigo from woad leaf material. The blue arrows point to the steps where oxidation forms indigo molecules (iii) and turns the cloth blue when lifted from a vat (v). The processes require controlling the oxygen gradient, high temperature and alkaline pH

Many historical woad processing and fermentation vat methods have been tested and reviewed by Hartl et al. [4]. In these methods, the maintenance of a dynamic balance between indigo formation and subsequent indigo reduction in traditional vats containing leaf material is required. Two somewhat separate microbially driven processes are used, one to extract indigo from the plant biomass and the other to reduce indigo and dye with it (Fig. 4).

Harvesting woad leaves is a challenge because the leaves cannot be stored as such. Therefore, indigo extraction may begin with the formation of ‘woad balls’. Dried woad balls can be stored for a long time and have been used in this form in trade since the Middle Ages. In short, the freshly collected woad leaves are crushed and squeezed into fist-sized balls and left to dry. However, in this way it is difficult to control the formation of indigo from its precursors [26]. The formation of indigo in the woad balls is reviewed by Kokubun et al. [40]. Inside the ball, plant and bacterial enzymes (oxygenases and hydroxygenases) hydrolyse isatan B into glucose (energy source for bacteria) and the indoxyl moiety. Inside the ball, bacterial respiration limits the entry of oxygen, which drives the slow dimerization of indoxyls to indigo. The question is, if acid-producing fermentation occurs, can the low pH preserve the isatan B molecules? In this way, indigo is formed within the leaf tissue from where it is difficult to extract [22]. It is important to limit the entry of oxygen into the system, as this would promote the composting of the leaf material and ultimately the degradation of indigo by bacteria such as *Bacillus pumilus* and *B. licheniformis* in woad vats [20], and some bacteria of the family Alcaligenaceae in the case of Japanese indigo [33]. The process may continue with couching, where the woad balls are crushed and fermented in moist piles or immersed in warm water (55 °C) for several days [4]. Couched woad turns into a dark, tarry mass. The purpose of this process may have been to break the leaf structure, which helps to release indigo from the leaf tissue in a vat [40].

Woad dyeing begins by crushing the woad balls and immersing them in warm water (> 50 °C), which then releases the indigo molecules into the water. It is possible that bacteria also contribute to the formation of indigo in the early stages of the vat itself. For example, the glycoside hydrolase enzyme from *Paenibacillus* sp. can cleave indican, and *Alcaligenes faecalis* uses sugars from indole as its sole carbon source [20]. Aerobic bacteria, such as Bacillaceae, consume oxygen from the stagnant water and their activity further increases the temperature of the vat. The woad vat soon develops a foul odour of sulphides (dimethylsulphides and methanediols), typical of bacteria degrading cruciferous plant material under anoxic conditions. The traditional vat can be maintained for several weeks or even months by feeding it with woad leaf material or extracted indigo powder and carbohydrates (such as bran) for the bacteria. Gradually, fermentative bacteria convert complex substrates into simple carbohydrates that can be used as food by indigo-reducing bacteria. In anoxic vats, for example, *Clostridium isatidis* can use indigo particles as electron acceptors and thus reduce indigo to its water-soluble leuco form (Fig. 3). Fermentation produces H^+^ ions and thus lowers the pH value of the vat, which must be adjusted to > 9 with sodium hydroxide [26]. The hot, anoxic and alkaline conditions in the vat change the complex composition of the bacterial community to select for thermophilic, fermentative, anaerobe and alkali-tolerant species [2]. The balance between facultatively anaerobic and obligately anaerobic bacterial communities in Japanese sukumo vats is important for the staining result [34]. Bacteria typical of the different developmental stages of the vat have been identified and are presented in Table 2.

### Biochemical Reactions for Indigo Reduction

*Clostridium isatidis* is one of the first characterised and most studied bacterial species able to reduce indigo. *C. isatidis* has even been recovered from archaeological material [4], probably as spores. Bacteria reduce the natural amorphous form of indigo more easily than the highly crystalline synthetic form. Smaller particles are more easily reduced than large particles [23]. Bacteria form a biofilm around particles of indigo and when the bacteria have reduced the particles, water-soluble leuco-indigo diffuses away. The bacteria, which can reduce indigo, have in common the ability to use insoluble electron acceptors and anaerobic metabolism of simple carbohydrates [26]. These bacteria often interact with solid particles through electrically conductive pilus-like nanowires [42]. *C. isatidis* may possess exoenzymes that interact with indigo particles to reduce their size, but the indigo-reducing protein itself is still speculative [23]. However, an azoreductase that uses NADH as an electron donor has been shown to reduce indigo [43].

The microbiome contains functional redundancy, which means that there are many potential indigo-reducing bacterial species, even if they represent only a small fraction of the microbial community of natural indigo dye vats (Table 2).

### Searching for the Origin of Indigo-Reducing Bacteria in a Vat

Where and how to introduce the right bacteria into the vat system remains a question. Microorganisms are naturally associated with the phyllosphere of the woad. Thus, bacteria in the traditional vat are introduced with the plant´s leaf material, and many of the bacteria may ultimately originate from the soil [34]. Bacteria and their spores can also enter the vat with washed leaves [20]. In addition, the couching of woad leaves introduces random bacteria into the material [3].

The presence of the right bacteria depends on their natural occurrence, and their enrichment and growth depend on proper maintenance of the system. For example, fermentative alkaliphilic bacteria are present in ubiquitous environments in Japan [29], but they must somehow be introduced into the vat with the leaf material. This highlights the importance of adequate pre-treatment of the original leaf material. Induction of favourable microbiota development is challenging, as a very small proportion of the required bacteria must be enriched in the original material [32]. Selection and enrichment of slow-growing, low-abundance bacteria can take months, as in sukumo, where an indigo-reducing bacterial community develops in Japan [26, 32, 33]. The decrease in redox potential required to initiate indigo reduction was improved by adding *Indigofera tinctoria* leaf powder in sukumo [34]. However, the authors conclude that the subsequent transition of the sukumo bacterial community was not influenced by the bacteria introduced with *I. tinctoria* leaf powder per se.

In a traditional vat, indigo reduction is initiated after a period when conditions become favourable [32]. Under anoxic conditions, alkaliphilic fermenting bacteria produce and maintain a reduction potential of − 600 mV, which is low enough for indigo reduction. Aino et al. [26] concluded that a change in oxidation–reduction potential triggers indigo reduction after enrichment of the appropriate bacterial community.

The functionality of the vat depends on the enrichment of indigo-reducing bacteria under carefully controlled conditions. However, the bacterial community in hot, anoxic and alkaline conditions is not stable, but bacterial dynamics change throughout the fermentation process and unfavourable bacteria can prevent the desired propagation of the vat process [32, 33]. There are several possible bacterial communities that can reduce indigo over long periods of time (reviewed in [2]). Differences between plant species and origin of leaf material, procedures and conditions (pH, temperature) contribute to different microbiomes in the traditional European and Japanese fermentation vats [2]. To date, more Japanese indigo-based sukumo vat bacteria have been named than woad vat bacteria (Table 2). Tu et al. [32] observed an increase in the proportion of *Alcalibacterium, Amphibacillus, Anaerobranca* and Bacillaceae in 5–7 days and *Polygonibacillus* in 4.5 months in Japanese-indigo vats.

Extracted indigo flakes lack sufficient bacteria population to initiate fermentation and indigo reduction in a new vat. Understanding the bacterial community transition associated with indigo formation and reduction helps to isolate functional strains that can lead to an indigo reduction process without harmful chemicals [20, 26]. Characterised indigo-reducing bacteria, such as *Clostridium isatidis*, are now available from culture collections [25]. However, the application of a single strain to the fermentation vat may be vulnerable to changing conditions and invasion of other bacteria from outside [31].

**Fig. 5:**
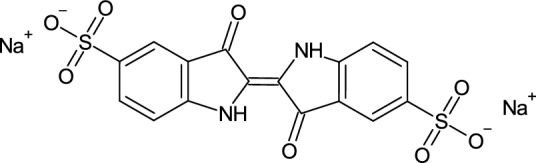
Indigotindisulfonate sodium (C_16_H_8_N_2_Na_2_O_8_S_2_) is chemical name of synthetic indigo carmine or shortly indigotine

New bacterial strains can be isolated and characterised using different isolation media, pH and oxygen levels and growth temperatures. Typically, complex high pH nutrient media are inoculated with diluted samples from vats and incubated anaerobically at 30 < 50 °C. Nutrient media may be such as standard reinforced clostridium agar (RCA) and contain peptones, yeast extract, sugars and salts. Sonicated indigo powder is added to the medium as an electron acceptor, but because indigo is not water soluble, its synthetic soluble derivative indigo carmine (Fig. 5) is often used instead [23].

Slow-growing bacteria, even in low numbers, can be outcompeted by facultative bacteria on nutrient agars. Therefore, special media containing vat ingredients have been developed to simulate environments such as indigo fermentation fluid. Hydrolysed composted Japanese indigo, sukumo, wheat bran and corn steep liquor have been used with indigo carmine and indigo powder in culture media [24, 31]. Indigo-reducing activity of isolated bacteria can be observed as a bright halo on dark medium or as the staining intensity of cotton fabric [26, 44].

## Woad in Rotation and Mixed Cropping

### Cultivation

Woad, *Isatis tinctoria*, is a member of the Brassicaceae family, along with many other important crop genera such as *Brassica, Camelina, Raphanus* and *Sinapis*. It is a biennial herb that overwinters as a rosette of leaves and flowers the following summer, after which it dies. Woad forms a two-layered root system with lateral roots in the upper soil and a deep taproot. Growing plants such as woad with deep taproots can improve soil quality, as they increase soil macroporosity and macropore diameter compared to fibrous roots [45]. This increase in macroporosity and macropore size can improve water infiltration, make the soil more permeable for plant roots and improve soil aeration.

Woad is not grown for food or fodder, but for its indoxyl-containing leaves and for seeds to ensure future generations of the dye plant. Flowering in the first year is an undesirable trait as it is detrimental to rosette development. High biomass production and rosette regrowth ability after leaf harvest ensure indigo yield [39]. Leaves are harvested 10 cm above the ground and allowed to regrow new ones before the next cut. The number of cuts depends on the length of the growing season. Early winter sowing and optimal plant density (10–20 plants m^−2^) influenced yield in south-eastern Spain [38]. Leaf biomass production varied widely (50–105 t fresh weight ha^−1^) depending on the number of harvests per season, and indigo yield also varied, ranging from 0.4 to 0.8 g kg^−1^ fresh leaf weight [38]. Woad is not a high nitrogen or water demanding crop and grows opportunistically on wasteland [10, 38]. Nitrogen fertilisation of 100 kg ha^−1^ before sowing and 50 kg ha^−1^ after each harvest was used in Spanish field trials [38]. Its growth requirements can also be met in northern latitudes, except for early sowing. Sales et al. [38] suggest transplanting as an alternative to early sowing.

Woad benefits from crop sequencing where it follows legumes, but it can have allelopathic effects on germination of other species, which may partly explain its success as an invasive weed in the western USA [46, 47]. Woad has been grown as part of in-row living mulch (mulch as an alternative to glyphosate) to control weeds in pear orchards in Slovenia [48]. Nevertheless, woad has been successfully grown as an effective nitrate scavenging intercrop in rows between leeks (*Allium porrum* [49]). Woad has good autumn growth and deep rooting, which may explain its success as a nitrate scavenging catch crop [50, 51]. Woad has been measured to contain nitrogen at 23.5 mg g^−1^ tissue and organic carbon at 410 mg g^−1^ tissue, with a C/N ratio of 17.45 [52].

### Woad’s Lack of Mycorrhizal Symbiosis

Cruciferous plants do not form symbiotic relationships with arbuscular mycorrhizal fungi (AMF), which normally help most other plants to obtain mineral nutrients. Instead, woad has been observed to form only rudimentary AMF [53, 54]. Therefore, two years of woad cultivation may result in lower numbers of AMF propagules in the soil compared to the cultivation of mycorrhizal plants [54]. It has been suggested that antimicrobial products derived from glucosinolates produced by cruciferous plants explain their inability to form AMF [55, 56]. However, normal growth of AMF has been observed near the roots of crucifer plants when compatible host plants are growing nearby. Therefore, a more likely explanation may be that cruciferous plants lack the necessary signalling molecules to communicate with the AMF [53].

### Functional Glucosinolates of Brassicaceae

In addition to indole derivatives, which may have potent anti-inflammatory activities, woad contains many other biologically active compounds as well, also in its roots [10, 37]. Characteristic group of compounds in cruciferous plant tissues are glucosinolates, which are secondary metabolites consisting of a glucose sugar moiety and nitrogen- and sulphur-containing aliphatic, aromatic or heterocyclic side chains (aglycones) derived from certain amino acids, e.g. tryptophan. Over 120 different glucosinolates have been identified in plants [57]. Their content and concentrations vary with plant species, developmental stage and plant tissue. Roots contain relatively more and a greater diversity of glucosinolates than shoots, and glucosinolate biosynthesis in roots is often constant [7]. The vegetative parts of a plant in the bud phase contain the most glucosinolates, and their biosynthesis is often induced, e.g. by aphid feeding [6, 7]. The degradation products of glucosinolates in turn give many Brassica crops their recognisable taste and smell, which are also utilised in condiments like horseradish paste or mustard. When plant tissues are damaged, glucosinolates encounter the plant cell wall enzyme myrosinase, which hydrolyses the molecules into glucose and several different S-containing products, depending on the side chain. The degradation products are often biologically active volatile compounds, thiocyanates, isothiocyanates (ITC) and nitriles [6, 58].

### Biofumigation Inhibits Nitrification and Repels Pests in Soil

Agricultural soils are sources of nitrous oxide (N_2_O) because they contain nitrogen (N) fertiliser or manure. Autotrophic aerobic microbes carry out ammonia oxidation (nitrification), whereas in poorly drained soils oxygen is depleted, creating conditions favourable for anoxic denitrification. Microbial ammonium oxidation or denitrification proceeds step by step, producing an intermediate, N_2_O, which is a potent greenhouse gas. Glucosinolate hydrolysis products, such as ITCs, can inhibit ammonia oxidation and reduce the number of nitrifying bacteria. Bending et al. [58] showed that 0.5 µg 2-propenyl-ITC g^−1^ soil dry weight inhibited nitrification, which is about 1% of the potential amounts formed after incorporation of Brassica crop residues or green manures into the soil. They refer to Elliot and Stowe [59] who measured woad releasing up to 4 µg of indole glucosinolates per g^−1^ fresh weight of root over 6 weeks. Woad leaves can contain 1.49 µmol g^−1^ tissue ITC-forming glucosinolates [52]. These analyses indicate the bioactive potential of woad in soil. The ecological importance of cruciferous crops is that within crop rotations, species and cultivars can be selected for their glucosinolate profiles and levels and used to manage N mineralisation from roots or crop residues, thus improving synchronisation with the needs of succeeding crops [58]. Elliot and Stowe [59] suggest that in the long term, ITCs may promote soil N mineralisation through a fumigant effect, whereby ITCs kill some of the biomass, which is subsequently degraded by surviving organisms, resulting in mineralisation of N from the dead cells.

Woad fresh leaf extracts showed significant inhibitory effects against bacteria [60], insects and the brown rot fungus *Coniophora puteana* [61] under test conditions. Woad may be one of the materials for biopesticides to replace synthetic ones.

## Conclusion

Due to rising input prices and interest in the environmental impact of primary production, woad can be considered an important alternative for farmers in crop rotation, as an intercrop or for revegetation of marginal and fallow land, if the soil is not too compacted [62]. Genotypes with high indigo yields have been identified for further breeding programmes in southern Europe [63]. Selecting plants for their secondary metabolites offers a possibility of breeding woad for its bioactive compounds, which further may reduce the need for synthetic chemicals. The number of AMF propagules may be reduced in woad field soils, but this has not been shown to be detrimental to mycorrhizal fungi or arbuscule formation in compatible crops. An interesting research question is, can woad root exudates even accelerate AMF formation or N-fixing nodulation of compatible inter-row plants? These biological processes could reduce the need for synthetic fertilisers.

Fresh woad leaves need to be processed soon after the harvest and pigment extraction requires containers, a lot of water and equipment to control temperature and pH. Woad harvesting and indigo extraction may therefore be most successful in cooperative arrangements. Energy-efficient on-farm extraction methods should also be developed further. The textile industry generates large amounts of wastewater and care must be taken to ensure that the production of plant dyes does not cause environmental problems because wastewater contaminated with indigo can be toxic to organisms in water and sediment [64].

The long history of indigo fermentation vats has created a cultural habitat that supports the enrichment of bacterial communities associated with different stages of the fermentation vat. The origin of the vat bacteria can be soil and plant material, and the bacteria in woad and Japanese indigo vats differ from each other. Many bacteria isolated from Japanese indigo vats have been given the species name *indireducens*. The name may reflect the rapid evolution of bacteria that have lived in traditional vats for many generations. Not many woad indigo reducers are as well-known as *Clostridium isatidis*, but sequencing of microbial communities has revealed greater diversity. The growth conditions of characterised bacterial inoculants from local sources can be optimised and the reduction process scaled up to at least medium- to pilot-scale production ventures [12]. Although the consumption of natural indigo is barely 1% of the total indigo used in markets today, its production is expected to continue as an alternative to synthetic indigo production and dyeing [3, 12]. Woad-derived indigo yields vary due to changes in precursor levels in the leaves, extraction efficiency and is not as pure as synthetic indigo. However, conscious consumers often appreciate the unique hue of natural dyes.

## References

[1] Niinimäki K, Tommila N, Räisänen R (2023) Colour collaboration. In: Niinimäki K et al (eds) Biocolours. Sustainable stories from nature, lab and industry. Aalto, Otava, p 311

[2] Aino K, Hirota K, Okamoto T, Tu Z, Matsuyama H, Yumoto I (2018) Microbial communities associated with indigo fermentation that thrive in anaerobic alkaline environments. Front Microbiol 9:2196. 10.3389/fmicb.2018.02196 30279681 10.3389/fmicb.2018.02196 PMC6153312

[3] Blackburn RS, Bechtold T, John P (2009) The development of indigo reduction methods and pre-reduced indigo products. Color Technol 125:193–207. 10.1111/j.1478-4408.2009.00197.x

[4] Hartl A, Gaibor ANP, van Bommel MR, Hofmann-de Keijzer R (2015) Searching for blue: experiments with woad fermentation vats and an explanation of the colours through dye analysis. J Archaeol Sci 2:9–39. 10.1016/j.jasrep.2014.12.001

[5] McDanell R, McLean AE, Hanley AB, Heaney RK, Fenwick GR (1988) Chemical and biological properties of indole glucosinolates (glucobrassicins): a review. Food Chem Toxicol 26(1):59–70. 10.1016/0278-6915(88)90042-7 10.1016/0278-6915(88)90042-7 3278958

[6] Agerbirk N, De Vos M, Kim JH, Jander G (2009) Indole glucosinolate breakdown and its biological effects. Phytochem Rev 8:101–120. 10.1007/s11101-008-9098-0

[7] Van Dam NM, Tytgat TO, Kirkegaard JA (2009) Root and shoot glucosinolates: a comparison of their diversity, function and interactions in natural and managed ecosystems. Phytochem Rev 8:171–186. 10.1007/s11101-008-9101-9

[8] Haramoto ER, Gallandt ER (2004) Brassica cover cropping for weed management: A review. Renew Agr Food Syst 19(4):187–198. 10.1079/RAFS200490

[9] Brown J, Morra MJ (2005) Glucosinolate-containing seed meal as a soil amendment to control plant pests 2000-2002. Subcontract Report NREL/SR-510-35254. https://docs.nrel.gov/docs/fy05osti/35254.pdf

[10] Speranza J, Miceli N, Taviano MF, Ragusa S, Kwiecień I, Szopa A, Ekiert H (2020) *Isatis tinctoria* L. (Woad): a review of its botany, ethnobotanical uses, phytochemistry, biological activities, and biotechnological studies. Plants 9:298. 10.3390/plants9030298 32121532 10.3390/plants9030298 PMC7154893

[11] Wenner N (2017) The production of indigo dye from plants. Fibershed Local fiber, local dye, local labor. p 47

[12] Linke JA, Rayat A, Ward JM (2023) Production of indigo by recombinant bacteria. Bioresour Bioprocess 10:20. 10.1186/s40643-023-00626-7 36936720 10.1186/s40643-023-00626-7 PMC10011309

[13] Fabara AN, Fraaije MW (2020) An overview of microbial indigo-forming enzymes. Appl Microbiol Biotechnol 104:925–933. 10.1007/s00253-019-10292-5 31834440 10.1007/s00253-019-10292-5 PMC6962290

[14] Bhushan B, Samanta SK, Jain RK (2000) Indigo production by naphthalene-degrading bacteria. Lett Appl Microbiol 31:5–9. 10.1046/j.1472-765x.2000.00754.x 10886605 10.1046/j.1472-765x.2000.00754.x

[15] Bidart GN, Teze D, Jansen CU, Pasutto E, Putkaradze N, Sesay AM, Fredslund F, Leggio LL, Ögmundarson O, Sukumara S, Qvortrip K, Welner DH (2024) Chemoenzymatic indican for light-driven denim dyeing. Nat Commun 15(1):1489. 10.1038/s41467-024-45749-3 38413572 10.1038/s41467-024-45749-3 PMC10899603

[16] Bechtold T, Burtscher E, Amann A, Bobleter O (1992) Reduction of dispersed indigo dye by indirect electrolysis. Angew Chem Int Ed Engl 31:1068–1069. 10.1002/anie.199210681

[17] Patra SK, Patra AK, Ojha P et al (2018) Vat dyeing at room temperature. Cellulose 25:5349–5359. 10.1007/s10570-018-1901-5

[18] Vuorema A (2008) Reduction and analysis methods of indigo. Doctoral thesis from the Department of Chemistry, University of Turku, Finland

[19] John P, Seymour K, Macias PG (2005) Production of natural indigo with a high purity. School of Plant Sciences. Project Spindigo

[20] Milanović V, Osimani A, Taccari M, Garofalo C, Butta A, Clementi F, Aquilanti L (2017) Insight into the bacterial diversity of fermentation woad dye vats as revealed by PCR-DGGE and pyrosequencing. J Ind Microbiol Biotechnol 44:997–1004. 10.1007/s10295-017-1921-4 28246965 10.1007/s10295-017-1921-4

[21] Minami Y, Kanafuji T, Miura K (1996) Purification and characterization of a β-glucosidase from *Polygonum tinctorium*, which catalyzes preferentially the hydrolysis of indican. Biosci Biotechnol Biochem 60:147–149. 10.1271/bbb.60.147

[22] Stoker KG, Cooke DT, Hill DJ (1998) An improved method for the large-scale processing of woad (*Isatis tinctoria*) for possible commercial production of woad indigo. J Agric Eng Res 71:315–320. 10.1006/jaer.1998.0329

[23] Nicholson SK, John P (2004) Bacterial indigo reduction. Biocatal Biotransform 22:397–400. 10.1080/10242420400024490

[24] Osimani A, Aquilanti L, Baldini G, Silvestri G, Butta A, Clementi F (2012) Implementation of a biotechnological process for vat dyeing with woad. J Ind Microbiol Biotechnol 39:1309–1319. 10.1007/s10295-012-1139-4 22581408 10.1007/s10295-012-1139-4

[25] Padden AN, Dillon VM, Edmonds J, Collins MD, Alvarez N, John P (1999) An indigo-reducing moderate thermophile from a woad vat, *Clostridium isatidis* sp. nov. Int J Syst Evol Microbiol 49:1025–1031. 10.1099/00207713-49-3-1025 10.1099/00207713-49-3-1025 10425759

[26] Aino K, Narihito T, Minamida K, Kamagata Y, Yoshimune K, Yumoto I (2010) Bacterial community characterization and dynamics of indigo fermentation. FEMS Microbiol Ecol 74:174–183. 10.1111/j.1574-6941.2010.00946.x 20695891 10.1111/j.1574-6941.2010.00946.x

[27] Hirota K, Okamoto T, Matsuyama H, Yumoto I (2016) Polygonibacillus indicireducens gen. nov., sp. nov., an indigo-reducing and oblicate alkaliphile isolated from indigo fermentation liquor for dyeing. Int J Syst Evol Microbiol 66:4650–4656. 10.1099/ijsem.0.001405 27503611 10.1099/ijsem.0.001405

[28] Hirota K, Nishita M, Tu Z, Matsuyama H, Yumoto I (2018) *Bacillusfermenti* sp., nov., an indigo-reducing oblicate alkaliphile isolated from indigo fermentation liquor for dyeing. Int J Syst Evol Microbiol 68:1123–1129. 10.1099/ijsem.0.002636 29458563 10.1099/ijsem.0.002636

[29] Nakajima K, Hirota K, Nodasaka Y, Yumoto I (2005) *Alkalibacterium iburiense* sp. nov., an obligate alkaliphile that reduces an indigo dye. Int J Syst Evol Microbiol. 10.1099/ijs.0.63487-0 16014476 10.1099/ijs.0.63487-0

[30] Park S, Ryu JY, Seo J, Hur HG (2012) Isolation and characterization of alkaliphilic and thermotolerant bacteria that reduce insoluble indigo to soluble leuco-indigo from indigo dye vat. J Korean Soc Appl Biol Chem 55:83–88. 10.1007/s13765-012-0014-3

[31] Nishita M, Hirota K, Matsuyama H et al (2017) Development of media to accelerate the isolation of indigo-reducing bacteria, which are difficult to isolate using conventional media. World J Microbiol Biotec 33:133. 10.1007/s11274-017-2300-z 10.1007/s11274-017-2300-z 28585166

[32] Tu Z, de Fátima Silva Lopes H, Hirota K, Yumoto I (2019) Analysis of the microbiota involved in the early changes associated with indigo reduction in the natural fermentation of indigo. World J Microbiol Biotechnol 35:123. 10.1007/s11274-019-2699-5 31346774 10.1007/s11274-019-2699-5

[33] Tu Z, de Fátima Silva Lopes H, Igarashi K, Yumoto I (2019) Characterization of the microbiota in long-and short-term natural indigo fermentation. J Ind Microbiol Biotechnol 46:1657–1667. 10.1007/s10295-019-02223-0 31432338 10.1007/s10295-019-02223-0

[34] Lopes HFS, Tu Z, Sumi H, Furukawa H, Yumoto I (2021) Indigofera tinctoria leaf powder as a promising additive to improve indigo fermentation prepared with sukumo (composted *Polygonum tinctorium* leaves). World J Microbiol Biotechnol 37:1–11. 10.1007/s11274-021-03142-y 10.1007/s11274-021-03142-y 34562162

[35] Daykin T (2011) Dissecting the indigo pathway. Thesis. Accessed from, https://api.semanticscholar.org/CorpusID:82200822

[36] Melanson D, Chilton MD, Masters-Moore D, Chilton WS (1997) A deletion in an indole synthase gene is responsible for the DIMBOA-deficient phenotype of bxbx maize. Proc Natl Acad Sci USA 94:13345–13350. 10.1073/pnas.94.24.13345 9371848 10.1073/pnas.94.24.13345 PMC24311

[37] Oberthür C, Schneider B, Graf H, Hamburger M (2004) The elusive indigo precursors in woad (*Isatis tinctoria* L.)–identification of the major indigo precursor, isatan A, and a structure revision of isatan B. Chem Biodivers 1:174–182. 10.1002/cbdv.200490009 17191785 10.1002/cbdv.200490009

[38] Sales E, Kanhonou R, Baixauli C, Giner A, Cooke D, Gilbert K, Arrillaga I, Segura J, Ros R (2006) Sowing date, transplanting, plant density and nitrogen fertilization affect indigo production from *Isatis* species in a Mediterranean region of Spain. Ind Crops Prod 23:29–39. 10.1016/j.indcrop.2005.03.002

[39] Spataro G, Negri V (2008) Adaptability and variation in *Isatis tinctoria* L.: a new crop for Europe. Euphytica 163:89–102. 10.1007/s10681-007-9604-2

[40] Kokubun T, Edmonds J, John P (1998) Indoxyl derivatives in woad in relation to medieval indigo production. Phytochem 49:79–87. 10.1016/S0031-9422(97)01069-8

[41] Garcia-Macias P, John P (2004) Formation of natural indigo derived from woad (*Isatis tinctoria* L.) in relation to product purity. J Agric Food Chem 52:7891–7896. 10.1021/jf0486803 15612772 10.1021/jf0486803

[42] Reguera G, McCarthy KD, Mehta T, Nicoll JS, Tuominen MT, Lovely DR (2005) Extracellular electron transfer via microbial nanowires. Nature 435:1098–1101. 10.1038/nature03661 15973408 10.1038/nature03661

[43] Suzuki H, Abe T, Doi K, Ohshima T (2018) Azoreductase from alkaliphilic *Bacillus* sp. AO1 catalyzes indigo reduction. Appl Microbiol Biotechnol 102:9171–9181. 10.1007/s00253-018-9284-y 30105570 10.1007/s00253-018-9284-y

[44] Padden AN, Dillon VM, John P, Edmonds J, Collins MD, Alvarez N (1998) *Clostridium* used in mediaeval dyeing. Nature 396(6708):225–225. 10.1038/24290

[45] Tang Y, Pan H, Zhang T, Cao L, Wang Y (2024) The dynamics of soil macropores and hydraulic conductivity as influenced by the fibrous and tap root systems. Agriculture 14:1676. 10.3390/agriculture14101676

[46] John P, Angelini LG (2009) Indigo—agricultural aspects. In: Bechtold T, Mussak R (eds) Handbook of natural colorants. John Wiley & Sons, Hoboken, p 4

[47] Monaco TA, Johnson DA, Creech JE (2005) Morphological and physiological responses of the invasive weed *Isatis tinctoria* to contrasting light, soil-nitrogen and water. Weed Res 45:460–466. 10.1111/j.1365-3180.2005.00480.x

[48] Paušič A, Tojnko S, Lešnik M (2021) Permanent, undisturbed, in-row living mulch: a realistic option to replace glyphosate-dominated chemical weed control in intensive pear orchards. Agric Ecosyst Environ 318:107502. 10.1016/j.agee.2021.107502

[49] Xie Y, Kristensen HL (2017) Intercropping leek (*Allium porrum* L.) with dyer’s woad (*Isatis tinctoria* L.) increases rooted zone and agro-ecosystem retention of nitrogen. Eur J Agron 82:21–32. 10.1016/j.eja.2016.09.017

[50] Aronsson H, Hansen EM, Thomsen IK, Liu J, Øgaard AF, Känkänen H, Ulén B (2016) The ability of cover crops to reduce nitrogen and phosphorus losses from arable land in southern Scandinavia and Finland. J Soils Water Conserv 71:41–55. 10.2489/jswc.71.1.41

[51] Munkholm LJ, Hansen EM (2012) Catch crop biomass production, nitrogen uptake and root development under different tillage systems. Soil Use Manag 28:517–529. 10.1111/sum.12001

[52] Brown PD, Morra MJ (2009) Brassicaceae tissues as inhibitors of nitrification in soils. J Agric Food Chem 57:7706–7711. 10.1021/jf901516h 19722704 10.1021/jf901516h

[53] Cosme M, Fernández I, Van der Heijden MG, Pieterse CM (2018) Non-mycorrhizal plants: the exceptions that prove the rule. Trends Plant Sci 23:577–587. 10.1016/j.tplants.2018.04.004 29753631 10.1016/j.tplants.2018.04.004

[54] Vestberg M, Palojärvi A, Pitkänen T, Kaipainen S, Puolakka E, Keskitalo M (2012) Neutral lipid fatty acid analysis is a sensitive marker for quantitative estimation of arbuscular mycorrhizal fungi in agricultural soil with crops of different mycotrophy. Agric Food Sci 21:12–27. 10.23986/afsci.4996

[55] Bressan M, Roncato MA, Bellvert F, Comte G, Haichar FEZ, Achouak W, Berge O (2009) Exogenous glucosinolate produced by *Arabidopsis thaliana* has an impact on microbes in the rhizosphere and plant roots. ISME J 3(11):1243–1257. 10.1038/ismej.2009.68 19554039 10.1038/ismej.2009.68

[56] Vierheilig H, Bennett R, Kiddle G, Kaldorf M, Ludwig-Müller J (2000) Differences in glucosinolate patterns and arbuscular mycorrhizal status of glucosinolate-containing plant species. New Phytol 146:343–352. 10.1046/j.1469-8137.2000.00642.x 33862976 10.1046/j.1469-8137.2000.00642.x

[57] Fahey JW, Zalcmann AT, Talalay P (2001) The chemical diversity and distribution of glucosinolates and isothiocyanates among plants. Phytochem 56:5–51. 10.1016/S0031-9422(00)00316-2 10.1016/s0031-9422(00)00316-2 11198818

[58] Bending GD, Lincoln SD (2000) Inhibition of soil nitrifying bacteria communities and their activities by glucosinolate hydrolysis products. Soil Biol Biochem 32:1261–1269. 10.1016/S0038-0717(00)00043-2

[59] Elliott MC, Stowe BB (1971) Distribution and variation of indole glucosinolates in woad (*Isatis tinctoria* L.). Plant Physiol 48:498–503. 10.1104/pp.48.4.498 16657825 10.1104/pp.48.4.498 PMC396893

[60] Fritz JI, Scheiner D (2015) Transformation and activity change of selected allelochemicals by microbial metabolism. J Allelochem Interact 1:39–56

[61] Seifert K, Unger W (1994) Insecticidal and fungicidal compounds from *Isatis tinctoria*. Z Naturforsch C 49:44–48. 10.1515/znc-1994-1-208 8148009 10.1515/znc-1994-1-208

[62] von Cossel M, Iqbal Y, Lewandowski I (2019) Improving the ecological performance of *Miscanthus* (*Miscanthus × giganteus* Greef et Deuter) through intercropping with Woad (*Isatis tinctoria* L.) and Yellow Melilot (*Melilotus officinalis* L.). Agriculture 9:194. 10.3390/agriculture9090194

[63] Angelini LG, Tozzi S, Nassi o Di Nasso NN (2007) Differences in leaf yield and indigo precursors production in woad (*Isatis tinctoria* L.) and Chinese woad (*Isatis indigotica* Fort.) genotypes. Field Crops Res 101:285–295. 10.1016/j.fcr.2006.12.004

[64] de Farias PMSG, de Jesus MB, dos Santos A, Freeman HS, Toukola P, de Albuquerque RR, Umbuzeiro GA (2025) Natural indigo toxicity for aquatic and terrestrial organisms. Ecotoxicol Environ Saf. 10.1016/j.ecoenv.2024.117606 10.1016/j.ecoenv.2024.117606 39733595

